# “Always learn today, stand ready to act tomorrow”—Po Tien: A pioneer of Virology in China

**DOI:** 10.1007/s13238-021-00870-7

**Published:** 2021-10-27

**Authors:** Xin Zhang, Huan Liu

**Affiliations:** 1 Institute of Biophysics, Chinese Academy of Sciences, Beijing, 100101 China; 2 Wuhan Institute of Virology, Chinese Academy of Sciences, Wuhan, 430071 China; 3 Chinese Center for Disease Control and Prevention, Beijing, 102206 China; 4 State Key Laboratory of Virology, Wuhan, 430072 China

**Figure 1:**
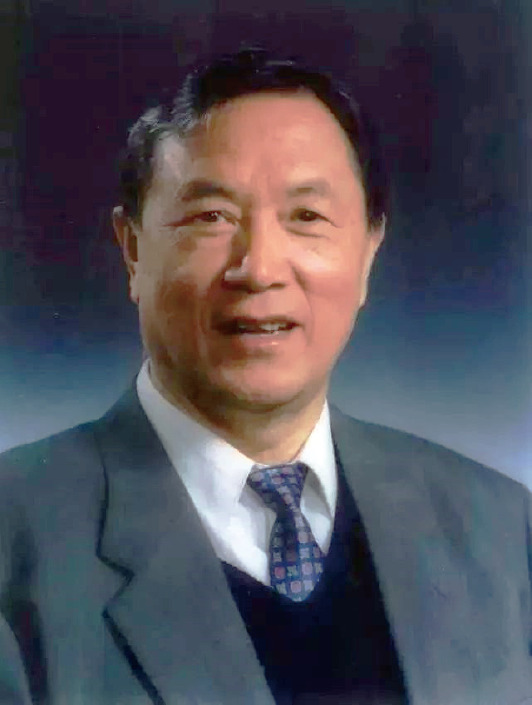
**Prof. Po Tien**

There is a scientist who was engaged in truly great achievements of modern virology in China. He first successfully applied viral satellite RNA as a biological control agent to prevent viral plant diseases (Tien, 1996), concluding that ribozyme-mediated high resistance against potato spindle tuber viroid in transgenic potatoes could reduce the pathogen to an undetectable level (Yang et al., 1997). He isolated a HBV-specific peptide associated with heat-shock protein gp96 from liver cancer tissues of HBV-infected patients for the first time (Meng et al., 2001). The Modern Virology Research Center and the first level 3 (P3) biosafety laboratory in China were established at Wuhan University under his guidance. He led his teams to develop an effective viral membrane inhibitor against SARS coronavirus infection at the outbreak of the epidemic in 2003**—**Po Tien (田波), a pioneer of modern virology in China (Fig. 1).

Prof. Po Tien (1931–2019) was a member of the Chinese Academy of Sciences (CAS), a full member of the American Society for Virology, and a member of the International Working Committee on Viroids. He was a principal investigator at the Institute of Microbiology, CAS, Professor at the College of Life Sciences, Wuhan University, and the Honorary Director of the Academic Committee of the Modern Virology Research Center, College of Life Sciences, Wuhan University.

Prof. Tien has been awarded many prizes for his great contributions to virology, including the State Natural Science Award and the State Science and Technology Progress Award two times, the CAS Natural Science Award and the CAS Science and Technology Progress Award five times, (Qing, 2020), and the title of Notable Asian Scientist conferred by *Asia Pacific Biotechnology News*.

Prof. Tien was born in Huantai, Shandong Province, in 1931. He witnessed the misery of Chinese people’s daily life in his childhood, and made up his mind to make a better world, which also contributed to his original aspiration of prioritizing serving the homeland in his life-long research career. Prof. Tien graduated from Beijing Agricultural University (now China Agricultural University) in 1954, after which he worked at the Institute of Microbiology, CAS. During his tenure, he was also a visiting scholar or guest professor at the University of Adelaide in Australia, University of Düsseldorf in Germany, University of Maryland and University of Wisconsin in the USA, as well as the Scottish Crop Research Institute in the UK. He was elected as a member of the CAS in 1991. In the early years of his research career, when one of the major problems in potato production in China was a degenerative disease, Prof. Tien set out to solve this problem. He analyzed the relationship between the local climate and potato degeneration, investigated virus-free potatoes, and the recovery of degenerated potatoes in Tibet. Potato degeneration was eventually controlled in China (Tien et al., 1982), which greatly facilitated the economic growth and social development. In 1981, he began to study biological control agents (BCA) based on satellite RNA, and found that satellite RNA could weaken viruses by inhibiting their genome replication. Two years later, he applied satellite RNA to prevent cucumber mosaic virus (CMV), the first successful case in the world (Tien, 1996). Since then, a series of greenhouse and field tests confirmed the effective disease control and high yields brought by this approach. These approaches for the prevention and control of plant viruses were met with great interest in China and internationally. By designing, cloning and transcribing a hammerhead ribozyme targeting the negative RNA strand of potato spindle tuber viroid (PSTVd) and a mutated nonfunctional ribozyme, Prof. Tien and his coworkers found that transgenic potatoes could effectively block the replication of PSTVd (Yang et al., 1997), and that the ribozyme-mediated high resistance of transgenic potatoes could reduce the amounts of potato spindle tuber viroid RNA to an undetectable level.

For national needs, in his 60s, Prof. Tien focused on health issues in China, from research on plant viruses to human viruses. By using direct peptide isolation from purified gp96 and microsequencing, his team successfully isolated an HBV-specific peptide associated with heat-shock protein gp96 from HBV-infected liver cancer tissues, and showed that a virus-specific peptide was bound to gp96 derived from liver tissues of patients with HBV-induced hepatocellular carcinoma (Meng et al., 2001), which laid a solid theoretical foundation for the development of engineered tumor vaccines against hepatocellular carcinoma and chronic HBV infection, as well as drugs for chronic hepatitis B and liver cancer. To study the drug resistance of HIV-1 and the efficiency of first-line highly active antiretroviral therapy (HAART) regimens consisting of generic nucleoside and non-nucleoside reverse transcriptase inhibitors in patients living in rural areas of Hubei Province, his team conducted two cross-sectional studies and firstly reported HIV-1 drug resistance after 2 years of HAART, which had implications for the implementation of HAART in developing countries (Luo et al., 2009). By studying the complex assembly of heptad repeat regions (HR1 and HR2) and the inhibition of viral fusion, his team found that both HR1 and HR2 of human respiratory syncytial virus F protein strongly inhibited virus fusion and formed a stable six-helix bundle *in vitro* (Wang et al., 2003).

**Figure 2:**
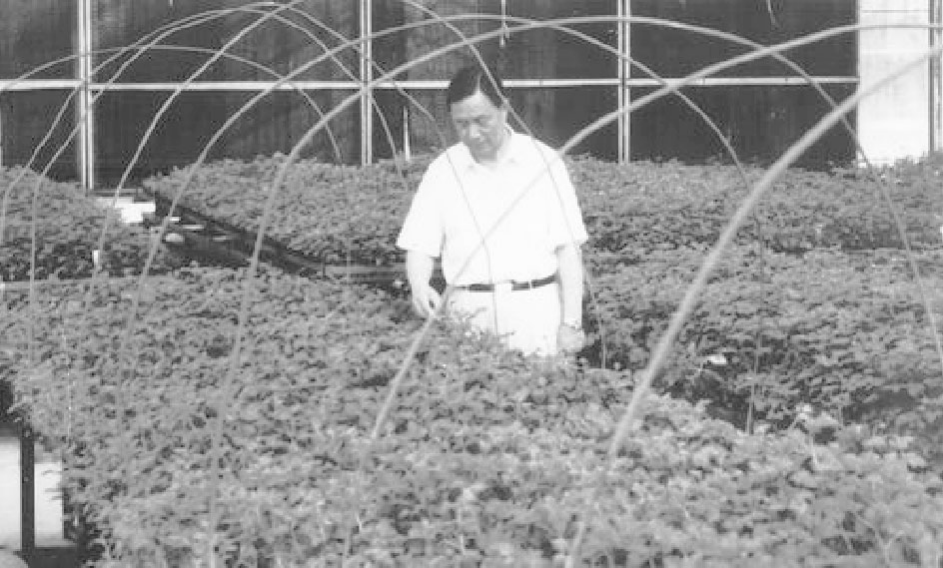
**Prof. Po Tien in the field**

**Figure 3:**
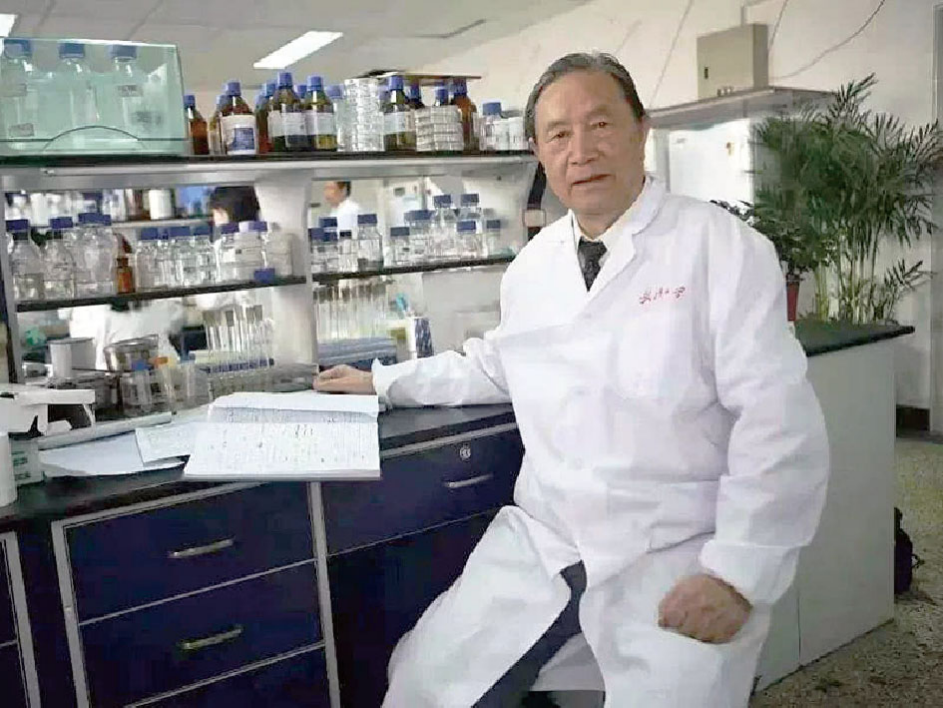
**Prof. Po Tien in the laboratory**

Prof. Tien served as a Distinguished Professor from 2001, and established the Modern Virology Research Center at Wuhan University. During the same period, the first P3 Laboratory was set up, and Prof. Tien proposed that a global vision and ambitious goals should be integrated into virology, which exerted a great positive influence on the development of virology in China. A large number of outstanding young and middle-aged overseas scholars were introduced to China, and cooperative relations with notable virological laboratories at home and abroad were also established. At the beginning of the SARS epidemic in 2003, Prof. Tien was immediately aware of the necessity and urgency of the scientific research on SARS coronavirus. Taking advantage of the P3 biosafety facility established in 2001, he led research teams investigating the fusion mechanism of SARS coronavirus and its inhibitors, and discovered SARS coronavirus nonstructural protein nsp14 as a novel cap N7 methyltransferase (Chen, 2009), which paved the way for the screening of anti-SARS drugs. The Ministry of Science and Technology issued a guideline on establishing a State Key Laboratory in the field of infectious diseases in 2004 (Fig. 2). Under the suggestion and guidance of Prof. Tien, Wuhan University and Wuhan Institute of Virology, CAS jointly applied for the State Key Laboratory of Virology, which was approved one year later. The State Key Laboratory of Virology is a world-renowned institution with a significant capacity for innovation in virology, forming a key component of the national scientific and technological strategic infrastructure (Guo et al., 2011). The State Key Laboratory of Virology will continue to play an important role in building Healthy China (Fig. 3).

**Figure 4:**
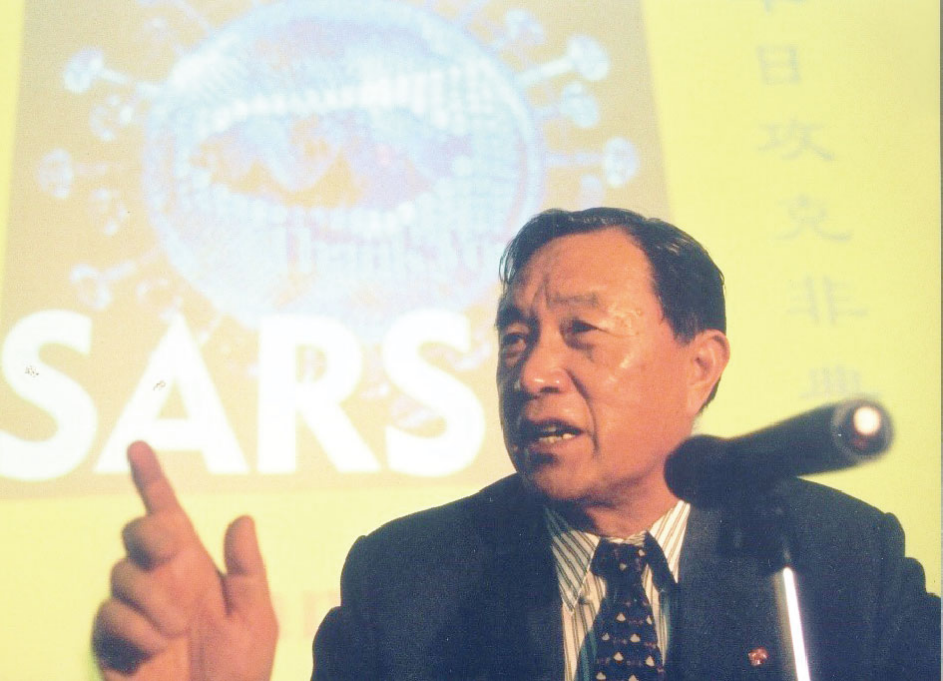
**Prof. Po Tien fighting against the SARS epidemic on the front line**

After witnessing the atrocities of war in childhood and experiencing hardship in his youth, Prof. Tien determined to serve the nation and the people all his life. When China suffered from low food production, he worked hard to study plant diseases to meet the people’s needs and fight hunger. As the country grew stronger, he strived to solve life and health issues, and thereby improved public health and quality of life. Prof. Tien not only made outstanding contributions to the development of virology, but also cultivated many excellent young talents, enabling for further progress of science and technology. “Only by thinking about the development of this discipline in depth could a virologist reflect on this issue at a high point.”, said Prof. Tien. As a prominent scientist, Prof. Tien was renowned for his determination for lifelong learning, and his vision of serving the country and its people. He has made great contributions to the development of virology in China by conducting in-depth research and developing the field, standing out through his focus on cutting-edge international scientific issues, and fighting for major strategic needs of the country. We will always be inspired by his scientific spirit of “Always learn today, stand ready to act tomorrow” (Fig. 4).
